# Corneal Regeneration Using Adipose-Derived Mesenchymal Stem Cells

**DOI:** 10.3390/cells11162549

**Published:** 2022-08-16

**Authors:** Jorge L. Alió del Barrio, Ana De la Mata, María P. De Miguel, Francisco Arnalich-Montiel, Teresa Nieto-Miguel, Mona El Zarif, Marta Cadenas-Martín, Marina López-Paniagua, Sara Galindo, Margarita Calonge, Jorge L. Alió

**Affiliations:** 1 Cornea, Cataract and Refractive Surgery Unit, Vissum (Miranza Group), 03016 Alicante, Spain; 2 Division of Ophthalmology, Universidad Miguel Hernández, 03550 Alicante, Spain; 3 IOBA (Institute of Applied Ophthalmobiology), University of Valladolid, 47011 Valladolid, Spain; 4 CIBER de Bioingeniería, Biomateriales y Nanomedicina, Instituto de Salud Carlos III, 28029 Madrid, Spain; 5 Castile and Leon Networking Center for Regenerative Medicine and Cell Therapy, 47011 Valladolid, Spain; 6 Cell Engineering Laboratory, La Paz University Hospital Health Research Institute, IdiPAZ, 28046 Madrid, Spain; 7 IRYCIS, Ophthalmology Department, Ramón y Cajal University Hospital, 28034 Madrid, Spain; 8 Cornea Unit, Miranza IOA, 28003 Madrid, Spain; 9 Department of Cell Biology, Histology and Pharmacology, University of Valladolid, 47002 Valladolid, Spain; 10 Optica General, Saida 1600, Lebanon

**Keywords:** stem cells, adipose-derived stem cells, regenerative medicine, corneal transplant, corneal regeneration, decellularized cornea, cornea, corneal stroma, corneal epithelium, cellular therapy, mesenchymal stem cells, extracellular vesicles

## Abstract

Adipose-derived stem cells are a subtype of mesenchymal stem cell that offers the important advantage of being easily obtained (in an autologous manner) from low invasive procedures, rendering a high number of multipotent stem cells with the potential to differentiate into several cellular lineages, to show immunomodulatory properties, and to promote tissue regeneration by a paracrine action through the secretion of extracellular vesicles containing trophic factors. This secretome is currently being investigated as a potential source for a cell-free based regenerative therapy for human tissues, which would significantly reduce the involved costs, risks and law regulations, allowing for a broader application in real clinical practice. In the current article, we will review the existing preclinical and human clinical evidence regarding the use of such adipose-derived mesenchymal stem cells for the regeneration of the three main layers of the human cornea: the epithelium (derived from the surface ectoderm), the stroma (derived from the neural crest mesenchyme), and the endothelium (derived from the neural crest cells).

## 1. Introduction: Adipose-Derived Mesenchymal Stem Cells (ADSC)

Human adipose tissue-derived adult mesenchymal stem cells (ADSC) can be easily obtained from low invasive liposuction aspirates rendering a high number of multipotent stem cells unlike different sources including bone marrow mesenchymal stem cells (BM-MSCs) and dental pulp stem cells (DPSCs). ADSC exert their action by different mechanisms: (a) transdifferentiation: ADSC have the potential to differentiate into several lineages and to integrate into the regenerated tissue [1,2], and in the cornea [3]; (b) by a paracrine action promoting the regenerative processes through trophic factors secretion: ADSC secrete paracrine factors such as platelet-derived growth factor (PDGF), vascular endothelial growth factor (VEGF), hepatocyte growth factor (HGF), and transforming growth factor beta 1 (TGFβ1) [4]. These factors are known to induce neovascularization. Although neovascularization is very advantageous in several ischemic environments such as renal or cardiac disease [5,6], in the avascular cornea it can abrogate the corneal immune privilege and be implicated in corneal transplant rejection risk [7]. It has been demonstrated that ADSC also secretes IL-6 and IL-8 [7], which also increase neovascularization, but in a previous study (in which the cornea was damaged with a laser) we demonstrated that human ADSC injected into the rabbit corneal stroma for regenerative purposes did not induce a rejection response [3], suggesting that the effect of ADSC therapy is also dependent on the environment; and c) by suppressing the inflammation and immune reaction signalling [8]: In fact, their immunomodulatory properties are being increasingly studied for the treatment of several disorders [4], and the therapeutic effects of their secretome on several diseases, including corneal transplantation [7]. Moreover, in a model of corneal chemical burns, mesenchymal stem cells (MSCs) engrafted in the corneal stroma decreased inflammation allowing for epithelial regeneration by a drop in the secretion of IFN-γ and IL-2 and increased levels of TGF-β, IL-10 and IL-6 in the injured corneas [9]. In one of our recent studies, ADSC cultured in three different media, one standard medium and two media used to culture limbal stem cells for clinical application (CnT30 containing FGF and EGF among other undisclosed growth factors and SHEM, containing EGF), showed that different media changed their paracrine secretion exerting different paracrine effector functions in vivo in a model of chemical burn and in response to a novel model of corneal inflammation by alkali treatment in vitro [10]. SHEM cultured ADSC showed a better anti-inflammatory profile, decreasing their secretion of VEGF, MMP-2, IL-6 and TGF-β after 72 h of exposure to the conditioned media of alkali-treated corneal epithelial cells [10]. In vivo, corneas treated with amniotic membrane without cells decreased MMP-2 and IL-6 expression. Further, corneas treated with control ADSC showed reduced expressions of TNF-α, MMP-2, IL-6 and MCP-1. Corneas treated with ADSC cultured in CnT30 or SHEM media down-regulated the expression of TNF-α, IL-6, MMP-2, MCP-1 and VEGFA. Interestingly, only the corneas treated with EGF-containing SHEM-media ADSC increased IL-10 expression. In the cornea, direct treatment of the corneal stroma or corneal epithelium with MSC exosomes could potentially achieve the same benefits of cellular therapy, but without providing the cellular component itself [11,12].

In vitro ADSC differentiation into the different corneal cell lineages have been achieved (Table 1):

In the case of ADSC in vitro differentiation into corneal epithelial and limbal stem cells, there has been shown that ADSC shows a basal expression of corneal epithelial cell markers such as ABCG2, p63, CK12, and CK16 [13], and has been shown to differentiate into them in response to specific media [14,15,16,17,18,19]. Specifically, ADSC cultured in the presence of EGF and KGF show corneal epithelial markers [15]. ADSC can thus be used as a source for the regeneration of the ocular surface [20]. However, in DMEM, CnT30 or SHEM, some authors report that the expression of these and other limbal markers, such as p63 or ABCG2, is lost during culture time indicating that corneal limbal phenotypes were not achieved [10].

With respect to ADSC in vitro differentiation into corneal stromal cells, ADSC is also able to differentiate into functional keratocytes in vitro and in vivo [3,21,22], specifically by exposure of ADSC to FGF2 together with co-culture with keratocytes. Several authors, including reports from our research group, have demonstrated that these stem cells can not only survive and differentiate into adult human keratocytes but are able to express collagens type I, type VI and keratocan without inducing an immune or inflammatory response [3,23,24].

As for ADSC in vitro differentiation into corneal endothelial cells, recently, we adapted several differentiation methods previously used for induced pluripotent stem cells (iPSCs) and embryonic stem cells (ESC) and achieved the directed differentiation of human ADSC to corneal endothelial cells (CECs). Using a two-step process by a previous differentiation into the neural lineage and then further differentiation into CECs. Specifically, the first step uses a dual Smad inhibitor medium, containing Noggin and SB431542 together with FGF2, or CHIR99021 and SB431542 together with Heregulin β1, IGF1 and FGF2 as well. Then the second step for actual CEC differentiation includes a B27 supplement PDGFBB and DKK-2. Long-term ADSC-derived CECs were positive for markers S100, Na^+^/K^+^ ATPase and ZO1. In our hands, the protocol by Ali et al. 2018 [25] was the best adapted to such differentiation in terms of efficiency, time, and financial cost; however, the protocol by Wagoner et al. 2018 [26] was the best for CEC marker expression. As for actual CEC differentiation, all combinations rendered high numbers of Na^+^/K^+^ ATPase-positive cells (at least 80% with each combination or protocol used). In our hands, the Ali protocol was superior, with higher quantitative efficiency (94% Na^+^/K^+^ ATPase-positive cells) [27]. It shall be reminded that Na^+^/K^+^ ATPase positivity, although the most commonly used marker, is not specific to corneal endothelial cell differentiation.

## 2. Corneal Epithelium ADSC Regeneration: Preclinical & Clinical Evidence

The aetiology of corneal epithelium damage is multifactorial and includes a wide variety of eye diseases [28]. One of the most prevalent ocular surface disorders, affecting between 5.5% and 35% of the world’s population, is dry eye disease (DED) [29]. This pathology was defined by the International “Dry Eye Workshop” in 2017 as a multifactorial disease in which inflammation and neurosensory abnormalities in the ocular surface play a crucial role [30]. Patients with DED experience varying harshness of symptoms and signs, such as irritation, pain, photophobia, redness, and blurred vision. Inflammation of the tear-producing tissues leads to an alteration in quantity (and mainly quality) of the tear film and a possible disruption of the corneal epithelium. This is clinically manifested as punctate superficial keratitis, epithelial defects, ulcers, potential infections, and even perforations in the most severe cases, causing disabling vision or total blindness in some patients [30,31]. However, one of the most severe pathologies affecting the corneal epithelium is limbal stem cell deficiency (LSCD). In this case, the pathology is end-stage morbidity resulting from a critical reduction and/or dysfunction of limbal epithelial stem cells, which are responsible for continuous corneal epithelium renewal. It is caused by a wide variety of ocular surface disorders, such as chemical, thermal, or mechanical injuries, immune-based disorders, congenital disorders, sequalae of multiple ocular surgeries, or infections. This leads to deficient regeneration of the ocular surface and eventually to persistent epithelial defects, secondary infections, neovascularization, and chronic inflammation. Any of these can result in corneal opacity, loss of vision, and chronic pain [32].

Currently, the scarcity of long-term effective therapies for DED and LSCD is a serious problem that frustrates patients and doctors who see how the disease progresses, affecting the patient’s quality of life. Therefore, researchers and scientists are making great efforts to develop new therapeutic strategies based on biological products and cell-based therapies with the aim of inducing corneal epithelial regeneration, decreasing inflammation, maintaining the functionality of ocular surface tissues (cornea, limbus, and conjunctiva), including tear-secreting glands, and thus restricting disease progression.

### 2.1. Preclinical Evidence

#### 2.1.1. ADSC for the Treatment of DED

DED is considered an inflammatory-based disease [30] characterized by the increase of reactive oxygen species levels and some inflammatory molecules and the decrease of anti-inflammatory molecules, growth factors, such as EGF, and antioxidant enzymes in the ocular surface [33].

Considering that MSCs are currently proposed as a cell therapy for many diseases, particularly those with inflammatory and immune-mediated components, and that inflammation is the principal pathogenic factor in DED, transplantation of MSCs can be considered an alternative therapeutic option. The well-known immunosuppressive and anti-inflammatory potential of these cells, and the important secretory capability of different bioactive molecules with trophic, paracrine, and immunomodulatory functions [34,35,36] make these cells promising for the treatment of DED.

Some experimental in vivo DED studies have been performed using different sources of MSCs or their derivatives, such as bone marrow [37,38,39,40,41], adipose tissue [42,43,44], or umbilical cord [45], as well as different administration routes, such as topical eyedrops, intraorbital injections in lacrimal glands, subconjunctival injections, and intraperitoneal and intravenous injections.

Regarding ADSC, this source of cells has been used in some studies as an alternative treatment for DED. The possibility of using these cells was confirmed by Park et al. [42] who demonstrated the safety of repeated local transplantation of allogeneic ADSC into the region of the lacrimal gland in dogs during a 9-week follow-up. No signs of ocular pain were noted throughout the study, and no differences were shown in the Schirmer test in ADSC-transplanted eyes. Moreover, significant histologic lesions or abnormalities in the cornea, sclera, uvea, or retina were not noted after transplantation. In addition, the study showed that ADSC transplantation suppressed the T-cell proliferative response in dogs [42].

In addition to safety, preclinical in vivo experiments have demonstrated a significant improvement in clinical signs after ADSC transplantation for treating DED in dogs [46]. This work showed that ADSC implantation in the lacrimal glands is easy and effective cell therapy, increasing tear production and reducing conjunctival hyperaemia and corneal opacity [43,44,46].

Additionally, ADSC therapy in experimental DED models decreased proinflammatory cytokines, such as IL-2, IFN-γ, IL-17, and MMP-2, increased the expression of some anti-inflammatory cytokines (IL-6, IL-10, TGF-β) and the regulatory T-cell balance (Th1/Th2), and regulated antiangiogenic factors, preventing progression of the process. In terms of regenerative effects, these cells increased the number of Meibomian glands, goblet cells, and aqueous tear volume, improving tear film stability and subsequent corneal healing [44,46,47,48].

Bittencourt et al. also compared the outcome of ADSC transplantation on canine eyes presenting with moderate versus severe DED signs [44]. This comparison revealed that normal tear production was achieved in moderate DED after ADSC transplantation up to the 1-year follow-up. However, in severely affected dogs, normal tear production was not reached.

**Figure 1:**
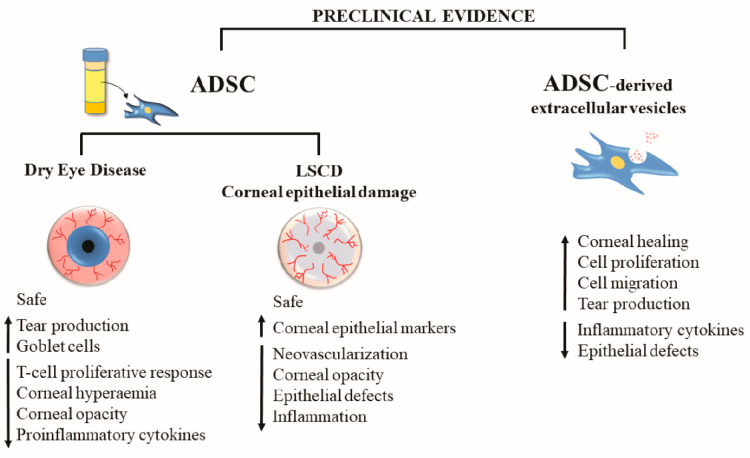
Preclinical evidence of the effects of ADSC-based therapy for the treatment of corneal epithelial damage (ADSC: adipose tissue-derived mesenchymal stem cells; LSCD: limbal stem cell deficiency).

Importantly, as it has been previously demonstrated in other pathologies, allogeneic ADSC transplantation is a safe and effective treatment, especially for moderate DED, and does not require lifelong medical care in animal models. Moreover, based on the several capabilities of ADSC, such as anti-inflammatory, immunomodulatory, antiangiogenic, tear production potential, reduction of neutrophil and macrophage infiltration, epithelial recovery, improvement of Meibomian glands, and increase of goblet cells, these cells can be considered a promising source of stem cells to treat DED (Figure 1) [43,47,48].

#### 2.1.2. ADASC for the Treatment of Limbal Stem Cell Deficiency and Corneal Epithelial Damage

Among the cell-based therapies currently available, the treatment of choice for LSCD is the transplantation of cultivated limbal epithelial cells (CLET) that can replace those that have lost their functionality [49,50]. However, the low availability of donors and the difficulty in culturing limbal epithelial cells [51] make MSCs an encouraging alternative to the treatment of LSCD [52,53].

Specifically, a promising MSC in the treatment of the ocular surface is obtained from adipose tissue (ADSC). ADSC administered by topical application [54], subconjunctival injection [55,56], and using amniotic membranes [57] or other substrates, such as contact lenses [58] or poly-L-lactic acid carriers [59] have been demonstrated to be safe. None of the animals showed any adverse events or toxicological effects after ADSC administration, even when xenogeneic transplantation was performed by administering human cells to the ocular surface of rats [54] and rabbit models [55,57,58]. Additionally, ADSC transplantation reduced clinical signs, such as neovascularization, corneal opacity, and epithelial defects, in experimental models of LSCD and corneal epithelial damage [54,55,57,58,59]. ADSC promoted a reduction of these clinical signs when they were differentiated in vitro to corneal epithelial cells and transplanted to the ocular surface in experimental models of corneal epithelial damage [17,60].

In addition, ADSC transplantation has been demonstrated to reduce inflammation of the ocular surface [54,57] by decreasing the infiltration of CD3+ T-cells and iNOS-expressing cells [59] (adaptive and innate immunity, respectively) in experimental models of corneal epithelial damage. Regarding the engraftment of the cells, Zeppieri et al. showed that ADSC topically administrated in a rat corneal burn model reached the corneal epithelium and the corneal stroma 3 days after the application of the cells [54]. Moreover, Galindo et al. pointed out the presence of ADSC in the limbal stroma 8 weeks after transplantation of the cells to the ocular surface using amniotic membranes as a carrier in an LSCD model developed in rabbits [57]. These authors also observed the expression recovery of the corneal epithelial markers cytokeratin (CK) 3 and E-cadherin and the limbal epithelial stem cell markers CK15 and p63 after ADSC transplantation to the ocular surface of an LSCD rabbit model [57]. According to their results, Lin et al. showed expression restoration of the epithelial cell surface markers connexin 43 and β-catenin after ADSC subconjunctival injection in a corneal burn animal model. However, they did not observe the recovery of E-cadherin and p63 expression [55]. Additionally, Holan et al. did not find a restoration of CK3 and CK12 expression after ADSC transplantation to the ocular surface of an LSCD model using poly-L-lactic acid carriers [59].

This preclinical evidence demonstrates that ADSC has a therapeutic effect on the regeneration of the corneal epithelium in experimental models of corneal epithelial damage and LSCD (Figure 1). However, the molecular mechanisms of ADSC-based tissue restoration are not yet fully understood; therefore, more coordinated preclinical and clinical studies are necessary.

#### 2.1.3. Cell-Free Therapy with ADSC-Derived Extracellular Vesicles

In recent years, the therapeutic potential of extracellular vesicles (EVs) secreted by cells has gained tremendous interest in the field of regenerative medicine. EVs are particles naturally released from the cell that is delimited by a lipid bilayer and cannot replicate [61]. They are normally classified by their biogenesis and size into three main subtypes: (1) exosomes (40–200 nm in diameter), originating from the intracellular budding of endosomes and released into the extracellular space; (2) microvesicles (50–1000 nm in diameter), arising from the budding of the cell membrane; and (3) apoptotic bodies (500–2000 nm in diameter), resulting from cell compartmentalization during programmed cell death [62].

EVs play an important role in intracellular communication through the diverse cargo that they transport, including proteins, ARNs, and lipids. These molecules are involved in maintaining tissue homeostasis and in a wide variety of biological functions, such as cell proliferation, migration, regeneration, and immunoregulation [63]. The specific EV cargo depends on the cell type of origin, and although most cells secrete EVs, greater amounts are produced by MSCs [64,65].

Accumulating evidence supports the idea that EVs derived from MSCs have many of the therapeutic effects shown in MSC treatment since they are involved in the paracrine properties of MSCs [64]. Cell-free therapies with MSC-derived EVs have several advantages over conventional MSC therapies: (1) they are safer since there is no need to transfer living cells that might be tumorigenic or possess damaged DNA; (2) the content of MSC-derived EVs can be modified by genetic engineering of MSCs and therefore EVs loaded with therapeutic factors can be produced while avoiding safety concerns [66]; and (3) EVs can be administered at higher doses and, therefore, delivered to injured tissues with greater effectiveness than conventional MSC treatments [64,67]. These features make MSC-derived EVs a very attractive cell-free therapeutic approach for the treatment of different diseases, including those affecting the ocular surface [68,69,70].

EVs secreted by MSC-derived from bone marrow, corneal stroma, human placenta, and induced pluripotent stem cells have been reported to accelerate corneal epithelial wound healing, decrease corneal epithelial defects, and reduce inflammatory cytokine production in animal models of corneal epithelial wound healing, corneal scarring, and DED associated with both Sjögren’s syndrome and graft-versus-host disease (GVHD) (GVHD-DED) [71,72,73,74,75,76,77].

ADSC-derived EVs have been isolated not only from human cells but also from mouse, rat, pig, and rabbit cells. Their cargo has been mainly associated with biological processes, such as cell proliferation, migration, inflammation, and apoptosis, thus supporting the beneficial effects observed in both in vitro and in vivo studies of diverse diseases [65,78]. In cell cultures, ADSC releases EVs into conditioned media [79]. Topical application of cell-free conditioned media from rat ADSC has been reported to accelerate corneal healing in a rat model of chemical corneal burn through the involvement of proliferation, migration, and differentiation of corneal epithelial cells. These effects were mediated by several growth factors and corneal repair-inducing mediators. This cell-free topical treatment showed an equivalent effect on corneal epithelial regeneration compared to the application of a topical solution containing MSCs [12].

Both human and mouse ADSC-derived EVs administered as eye drops have also shown therapeutic efficacy in mouse models of DED induced by either desiccating stress (hADSCs-derived extracellular vesicles inhibit NLRP3 inflammasome activation and dry eye) or benzalkonium chloride [80]. Topical ADSC-derived EV-treated mice showed decreased corneal epithelial defects, increased tear production, decreased goblet cell loss, and reduced inflammatory cytokines production by inhibiting NLRP3 inflammasome signalling pathway activation. Therefore, topical instillation ADSC-derived EVs may represent a novel potential therapeutic alternative for the treatment of DED [80,81].

Cell-free therapy with MSC-derived EVs and, more specifically, with ADSC-derived EVs has shown promising preclinical results for corneal epithelial regeneration. However, thus far, most of these therapeutic effects have only been observed in the preclinical stages, and further evaluations are still necessary to solve some present challenges before reaching large-scale clinical applications. For example, the specific mechanism of action of these EVs is still relatively unknown, and additional preclinical studies are needed to decipher which factors present in MSC-derived EVs are responsible for each specific therapeutic effect. Furthermore, EVs are very heterogeneous depending on the MSC source, and the therapeutic effect of EVs in eye diseases is associated with the origin of MSCs. In addition, it is essential to determine the optimum dose and route of administration to maintain the long-lasting effects of EVs in the eye.

### 2.2. Clinical Evidence

#### ADASC for Corneal Epithelium Regeneration

Over the last few years, the field of cell-based therapies to treat both DED and LSCD pathologies has undergone great development. Experimental and clinical MSC-based therapies have increased in the last decade due to their immunomodulatory and anti-inflammatory properties, their capability to migrate to injured and inflamed tissues, and their capacity to differentiate into corneal epithelial cells [82].

Currently, 12 clinical trials in which MSCs have been used as cell therapy to treat damaged corneal epithelium have been registered in the U.S. National Library of Medicine (*ClinicalTrials.gov*). Four of them propose using ADSC-based therapy to treat the damaged corneal epithelium of patients affected by different pathologies: DED associated with Sjögren’s syndrome (NCT04615455), aqueous-deficient DED (NCT03878628), keratopathy associated with bilateral LSCD (NTC01808378), and corneal ulcer/inflammatory dystrophy (NCT04484402). However, thus far, only one of these clinical trials has been published [83] (Table 2), reporting new and hopeful results about the safety and efficacy of ADSC-based therapy. Here, researchers reported an open-label, prospective, and phase I clinical trial in which a total of 7 patients affected by aqueous-deficient DED due to Sjögren’s syndrome were treated with a single dose of allogeneic ADSC (an average of 3.10 × 10^6^ ADSC/dose), administered directly into the lacrimal gland through transconjunctival injection. Patients were evaluated at 1, 4, and 16 weeks. Adverse events were registered at each visit, although none were related to cell-based therapy [83]. Symptoms (Ocular Surface Disease Index -OSDI) and signs (osmolarity, tear film breakup time -TBUT, Schirmer’s test, and tendency in corneal staining) improved [83]. The authors concluded that a single dose of ADSC injected into the lacrimal glands of patients affected by aqueous-deficient DED can improve their clinical signs and symptoms without adverse events.

Currently, no further clinical trials using ADSC as a cell therapy for the corneal epithelium have been published. However, two other clinical trials in which MSC-based therapy has been used to treat damaged corneal epithelium have been developed. BM-MSCs were used in both cases. One of these was carried out by Weng et al. in 2012, [84] (Table 2). A total of 22 patients affected by GVHD-DED were recruited. All were treated with allogeneic BM-MSCs administered by intravenous injection (mean dose of cells was 0.95 × 10^6^ BM-MSCs/Kg body weight) [84]. The final follow-up time was 3 months. Adverse events directly related to the treatment were not reported. In this case, the treatment improved the clinical symptoms in the patients (54.55%). The authors described a relationship between the increase in CD8+ and the improvements observed in DED symptoms (decrease in the OSDI test score) and signs (National Institute of Health consensus criteria and Schirmer’s test) [59].

**Figure 2:**
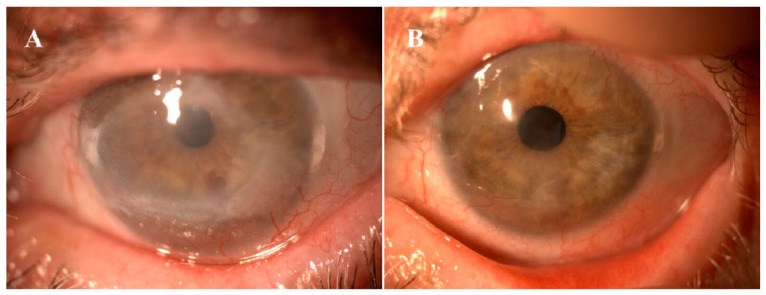
64-year-old female with chronic epithelial defect due to local chemotherapy for ocular surface neoplasia. Before (**A**) and 12 months after mesenchymal stem cell transplantation (**B**).

Finally, a prospective, randomized, double-masked clinical trial in phases I-II was developed by Calonge et al. to treat patients affected by LSCD with cell-based therapy [52] (Table 2). Here, allogeneic BM-MSC transplantation (MSCT) was compared with allogeneic cultivated limbal epithelial transplantation (CLET) to improve corneal epithelial damage. A total of 27 patients were recruited and 11 CLET and 17 MSCT were analyzed. Allogeneic cells (limbal epithelial cells or MSCs) were cultivated on a de-epithelialized amniotic membrane performing a complex of amniotic membrane cells that were then sutured to the sclera. A dose of 250,000 cells was administered. The follow-up time was 6 and 12 months after cell transplantation. Adverse events related to cell products were not reported. The corneal epithelial phenotype improved in patients treated with MSCT (71.4%) and CLET (66.7%) without significant differences. Global success was 76.5–85.7% for MSCT at 6–12 months without significant differences with CLET. These results suggest that MSCT improves the ocular surface affected by deficiency of limbal stem cells in a similar way to CLET (Figure 2) [52].

Recently, patients affected by GVHD-DED were treated with exosomes isolated from MSCs in a prospective clinical trial [77] (Zhou 2022, Table 2). Fourteen patients, with 28 eyes in total, were included in the trial. These patients showed an improvement in systemic GVHD signs with established treatments, but the ocular pathology was not improved. Patients were treated with 10 µg/50 µL of exosomes obtained from human umbilical cord-derived MSCs (UMSCs) that were administered as eye drops 4 times daily over 14 days. No adverse events related to cell products were reported. Symptoms (OSDI) and signs (TBUT, Schirmer’s test, and corneal staining) improved 14 days after treatment [77]. In addition, these authors suggested that exosomes reprogramme pro-inflammatory M1 macrophages towards immunosuppressive M2. These results showed remission of DED symptoms and an improvement in quality of life, suggesting that exosomes isolated from UC-MSCs can be a safe and innovative treatment for patients affected by GVHD-DED [77].

In summary, although several clinical trials have revealed the safety and efficacy of MSCs, such as ADSC, to treat damaged ocular surface epithelium by different pathologies, such as DED and LSCD, more preclinical and clinical studies are necessary to confirm these results before ADSC-based therapy may become an established treatment for these patients.

## 3. Corneal Stroma ADSC Regeneration: Preclinical & Clinical Evidence

### 3.1. Preclinical Evidence

The stroma constitutes more than 90% of the corneal thickness, and many features of the cornea, including its strength, morphology and transparency are attributable to the anatomy and properties of the corneal stroma. The corneal stroma is a collagen-rich extracellular matrix (ECM) assembled to provide its optical properties, basically transparency and refraction of light. Tightly packed parallel collagen fibrils are stacked in multiple lamellae which are regulated by a stromal extracellular matrix of a group of tissue-specific keratan sulfate proteoglycans [85]. Scattered between the lamellae there is a scarce population of keratocytes which remain quiescent throughout adult life [86]. Thus, unlike the regenerative capability of the corneal epithelium, the renewal of the corneal stroma is not based on a cycle of cell apoptosis and division, but on relatively slow collagen and ECM turnover mediated by the normal keratocytes that allow for proper lamellar alignment [87,88].

Many diseases such as corneal dystrophies, scars or ectatic disorders induce a distortion of its anatomy or physiology leading to loss of transparency and subsequent loss of vision. Enormous efforts have been put into replicating the corneal stroma in the laboratory to find an alternative to classical corneal transplantation, but this has still not been yet accomplished due to the extreme difficulty in mimicking the highly complex ultrastructure of the corneal stroma, obtaining substitutes that do not achieve either enough transparency or strength [89,90]. Moreover, synthetic scaffold-based designs have raised some important concerns such as strong inflammatory responses induced by their biodegradation, or chronic nonspecific inflammatory responses [91]. On the other hand, several corneal decellularization techniques have been described, which provide an acellular corneal ECM [92]. These scaffolds have gained attention in the last few years as, in contrast with synthetic scaffolds, they provide an ideal natural environment for the growth and differentiation of cells (either transplanted donor cells or migrating host cells) [23]. In addition, components of the ECM are generally conserved among species and are well tolerated by xenogeneic recipients.

In the last decade, cellular therapy of the corneal stroma using MSCs from extraocular sources has gained interest: previous studies show that MSCs are capable of differentiating into adult keratocytes in vitro and in vivo, and these stem cells not only survive and differentiate into adult human keratocytes in xenogeneic scenarios without inducing any inflammatory reaction [3,23], but also (I) produce new collagen within the host stroma [3,21], (II) modulate preexisting scars by corneal stroma remodelling [93,94], and (III) improve corneal transparency in animal models for corneal dystrophies by collagen reorganization, as well as in animal models for metabolopathies by the catabolism of accumulated proteins [95,96,97,98].

#### 3.1.1. Available Stem Cells for Corneal Stroma Cellular Therapy

As we have seen, MSCs can be obtained from many human tissues, including adipose tissue, bone marrow, umbilical cord, dental pulp, gingiva, hair follicle, cornea and placenta [99]. Existing scientific evidence shows that all types of MSCs have probably similar behaviour in vivo [100], and thus are able to achieve keratocyte differentiation and modulate the corneal stroma with immunomodulatory properties [101]. Corneal stromal stem cells (CSSCs) are a promising source for cellular therapy as the isolation technique and culture methods have been optimized and refined [102]; presumably, they show greater differentiation potential into keratocytes as they are already committed to the corneal lineage [103]. However, isolating CSSCs autologously is more technically demanding considering the small amount of tissue that they are obtained from. Furthermore, this technique still requires a contralateral healthy eye, which is not always available (bilateral disease). Therefore, these drawbacks may still limit its use in clinical practice.

As previously discussed, it is important to highlight that the therapeutic effect of MSCs in a damaged tissue is not always related to the potential differentiation of the MSCs in the host tissue, as multiple mechanisms might contribute simultaneously to this therapeutic action, for example, the already established secretion of paracrine growth factors capable of stimulating the host tissue, in which case the direct cellular differentiation of the MSCs might not be relevant [101,104,105].

#### 3.1.2. Corneal Stroma Regeneration by MSC Therapy Strategies without Scaffold

Corneal stroma MSCs implantation has been assayed and studied by direct intrastromal transplantation or after implantation from the ocular surface, intravenously and the anterior chamber where cellular migration within the stroma is to be expected. This cellular implantation without a carrier aims to remodel or generate new ECM within the corneal stroma.

**(A)**  **Ocular Surface Implantation of Stem Cells**

Surface implantation of MSCs is the optimal approach for ocular surface reconstruction and corneal epithelium/limbal stem cell niche regeneration. However, surface implantation of MSCs would still play a role in the prevention or modulation of anterior stromal scars after an ocular surface injury (like a chemical burn). As discussed previously, MSCs secrete paracrine factors that enhance corneal re-epithelialization and stromal wound healing [106]. Basu et al. suggested the delivery of MSCs using fibrin glue [102]: they resuspended CSSCs in a solution of human fibrinogen, and this was added onto a wounded ocular surface with thrombin on the wound bed. Using this application, they demonstrated the prevention of corneal scarring in the mouse model together with the generation of new stroma with a collagen organization indistinguishable from that of native tissue.

**(B)**  **Intrastromal Implantation of Stem Cells**

The differentiation of human ADSCs in functional human keratocytes was demonstrated in vivo, for the first time, in a previous study of our group using the rabbit as a model [3]. These cells, once implanted intrastromally, express not only collagens type I and VI (the main components of corneal extracellular matrix), but also keratocyte-specific markers such as keratocan or ALDH, without inducing an immune or inflammatory response. These findings were later reproduced and confirmed by other authors in several research papers [101]. Du et al. [97] reported restoration of corneal transparency and thickness in lumican null mice (thin corneas, haze and disruption of normal stromal organization) three months after intrastromal transplant of human CSSCs. They also confirmed that human keratan sulphate was deposited in the mouse stroma and the host collagen lamellae were reorganized, concluding that delivery of human CSSCs to the scarred human stroma may alleviate corneal scars without requiring surgery [97]. Very similar findings were reported by Liu et al. who utilized human UMSCs using the same animal model [98]. Coulson-Thomas et al. found that, in a mouse model for mucopolysaccharidosis, transplanted human UMSCs participate both in extracellular glycosaminoglycans (GAG) turnover and enable host keratocytes to catabolize accumulated GAG products [95].

Since the amount of new collagen produced in vivo by the implanted MSCs has been shown to be limited, the concomitant use of decellularized human corneal stroma lenticules as a carrier for the MSCs has been assayed in order to provide stronger support to the severely weakened cornea. In the past, our group explored the recellularization of such human lenticules with human ADSCs in the rabbit model [23], and subsequently in keratoconic patients as we will see later [100]. Transplanted human ADSCs survive and still differentiate into corneal keratocytes within such decellularized human corneal stroma lenticules implanted within the corneal stroma of the host, obtaining the complete integration of the implant in vivo.

**(C)**  **Anterior Chamber Injection of Stem Cells**

Demirayak et al. reported that BM-MSCs and ADSCs, suspended in phosphate-buffered solution (PBS) and injected into the anterior chamber after a penetrating corneal injury in a mouse model, are able to colonize the corneal stroma and increase the expression of keratocyte specific markers such as keratocan, with a demonstrated increase in keratocyte density by confocal microscopy [94]. Conversely, the possible side effects of this MSC injection into the anterior chamber for the lens epithelium and trabecullum are highly questionable as it may induce scarring and subsequent glaucoma. Considering this, the potential clinical use of this approach, in our opinion, may be limited.

**(D)**  **Intravenous Injection of Stem Cells**

Systemic use, by intravenous injection, of MSCs has also been tested. Intravenous injection of BM-MSCs in mice after an allograft corneal transplant was able to colonize the transplanted cornea and conjunctiva (inflamed ocular tissues) but not the contralateral ungrafted cornea, simultaneously decreasing immunity and significantly improving allograft survival rate [107]. Yun et al. recently reported similar findings with the intravenous injection of iPSC-derived MSCs and BM-MSCs after a surface chemical injury, where they observed that the corneal opacity, inflammatory infiltration and inflammatory markers in the cornea were markedly decreased in the treated mice, without significant differences between both MSC types [108]. In contrast, our group did not observe any benefit in corneal allograft survival and rejection rates after systemic injection of rabbit ADSC prior to surgery, during surgery, and at various times after surgery in rabbits with vascularized corneas [7].

#### 3.1.3. Cell-Free Therapy with MSC-Derived Extracellular Vesicles

As discussed previously, it has been shown that EVs isolated from the culture media of human CSSCs had similar immunosuppressive properties and also significantly reduced stromal scarring in wounded corneas in the animal model in vivo [74]. In consequence, it has been suggested that the mechanism of action of these EVs could be through the transference of microRNA to the host cells. This finding suggests that, for some diseases such as the prevention or reduction of corneal scars, MSC EVs may provide a non-cell-based therapy [11]. Zhang et al. suggested that EVs released by transplanted UMSCs in the diseased cornea are able to enter into host corneal keratocytes and endothelial cells and enhance their functions [109]. In their in vitro experiment using mucopolysaccharidosis VII mice, they discovered that UMSC-secreted EVs assisted in the recycling process of accumulated glycosaminoglycans (GAGS) in the lysosomes of diseased cells [95].

These findings open an exciting new field for research as the use of MSC EVs per se could overcome some of the limitations and risks associated with the use of live stem cell implantation (safety concerns, costs, strong regulatory requirements, difficulties in storage and delivery), given that EVs are inorganic compounds that can be potentially applied topically [68]. Optimized cell culture conditions coupled with bioengineered parental cell lines could offer an expandable source of high-potency EVs with low variation in quality [68].

### 3.2. Clinical Evidence

**Figure 3:**
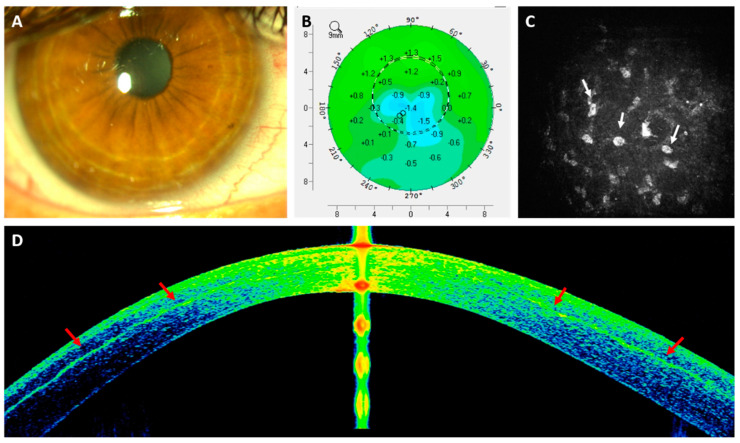
Autologous h-ADSCs corneal stroma implantation for advanced keratoconus. (**A**) Slit lamp picture 24 h after the procedure; (**B**) Topographic changes (Pentacam) between preop and 6 months after surgery. Observe the overall stability of the keratometric parameters; (**C**) Corneal confocal biomicroscopy pictures at the surgical plane in the first postoperative month. Stem cell survival is confirmed by the presence of cells showing a more rounded morphology (white arrows) (image corresponds to an area of 0.1 mm^2^); (**D**) AS-OCT picture 6 months postoperatively. Note the patched hyper-reflective areas (red arrows) at the level of the stromal pocket compatible with areas of new collagen production.

**Figure 4:**
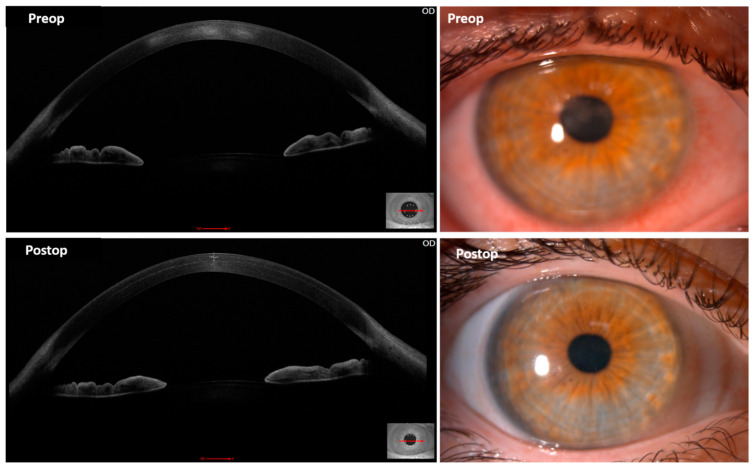
Anterior segment OCT and slit-lamp images from a keratoconus patient before (**up**) and 1 month after (**down**) autologous mid-stromal ADSC implantation. Observe the postoperative improvement in the density and severity of the central preoperative scars both clinically and in OCT. The ADSC intrastromal deposit of new collagen can be already observed 1 month after surgery in this case (**down-left image**).

To the best of our knowledge, the only published clinical data using stem cells for the regeneration and treatment of the human corneal stroma is from a previous study by our group [110,111,112,113]. In it, we implanted a suspension containing quiescent autologous ADSCs (obtained by elective liposuction) in a mid-stroma femtosecond laser-assisted lamellar pocket in patients with advanced (stage IV) keratoconus with poor visual function and already candidates for corneal transplantation. None of these cases had previous corneal surgeries or visually significant corneal scars. We could confirm, in humans, all previous evidence reported in the animal model: ADSCs survival in vivo, perfect biointegration without tissular or clinical inflammatory response, neocollagen production (about 15 µm thick on average) (Figure 3 and Figure 4), together with a moderate efficacy in terms of visual improvement (about 2 lines gain) [111,112,113]. Corneal keratometry readings and subjective refraction did not experience relevant changes in the long-term observation.

According to this clinical and all pre-clinical available evidence, the direct intrastromal implantation of MSC within the cornea achieves the production of new ECM, but it is not expected to be quantitatively enough in order to be able to restore the thickness of a severely diseased human cornea (like in extreme keratoconic eyes). However, the direct injection of stem cells may provide a promising treatment modality for corneal dystrophies, corneal stroma progressive opacification in the context of systemic metabolic disorders, and the modulation of corneal scars. Actually, one patient in our trial showed a clinical improvement of previously established paracentral scars (Figure 4) [111], and the study of the corneal stroma optical density of those patients implanted with autologous ADSC demonstrated a significant decrease in the anterior stroma densitometry up to 36 months postop, what supports the evidence already observed in the animal model that MSC enhance corneal stroma remodelling and collagen turn-over with the potential of improving established scars (*Corneal Stroma Densitometry Evolution in a Clinical Model of Cellular Therapy for Advanced Keratoconus. TVST. Epub ahead of print*). We could also observe by confocal microscopy, that this stroma densitometric improvement occurs in parallel with a gradual and significant increase in the keratocyte cellularity within the anterior, mid and posterior stroma of these patients [114]. In summary, implanted ADSC produce the deposit of new ECM, and probably mediated by paracrine secretion through EV’s stimulate the keratocyte population, which enhances the corneal stroma remodelling, which is finally linked to the improvement of its optical transparency. While SC-free therapy with MSC EVs could potentially achieve a similar therapeutic effect, the in vivo production of ECM probably still requires the implantation of the cellular component. However, it remains unclear the clinical relevance of such deposits and further in vivo biomechanical studies are necessary to clarify if these new collagen fibres have any influence on the corneal stiffness and so possess the capacity to halt the natural progression of the keratoconic disease.

In severely thinned corneas, MSC therapy can be assisted with the use of a lamina of decellularized corneal stroma as a carrier for the MSCs. In contrast with the isolated implantation of autologous ADSC, the combined approach (8.5 mm in diameter and 120 microns thick lenticule of human decellularized cornea stroma colonized with autologous ADSCs) is able to obtain a real improvement in the refractive sphere through a significant keratometric flattening, together with an improvement on the corneal aberrations and a subtotal restoration of the corneal thickness [110,112,113]. This decellularized tissue didn’t induce any inflammatory reaction in our study sample, and biointegrated well within the host corneal stroma. The addition of ADSCs was demonstrated to enhance the host keratocyte colonization of such implanted tissue [110,112,113].

Prasad Eye Institute in India is currently conducting the first clinical trial using human CSSCs (https://clinicaltrials.gov/ct2/show/NCT03295292 (accessed on 15 July 2022) for the treatment and prevention of corneal scarring in severe inflammatory corneal surface pathologies such as burns, ulcers and scars. In this trial, they are assessing the efficacy and safety of the treatment with 0.5 million cultured allogeneic human CSSCs, obtained from cadaveric corneoscleral rims, and delivered to the ocular surface embedded into a fibrin sealant gel. Two years follow-up preliminary results suggest encouraging outcomes in restoring corneal transparency and vision, showing statistically better corrected visual acuity, corneal clarity (evaluated both clinically and with scheimpflug imaging) and lesser corneal vascularization than control patients. While 60% of control eyes required a second surgical intervention, only 13.3% of the experimental eyes did (unpublished data, presented in ARVO 2019 Annual Meeting).

## 4. Corneal Endothelium ADSC Regeneration: Preclinical Evidence

The corneal endothelium is a monolayer coating the inner surface of the cornea. Its main role is regulating corneal hydration, which is key to keeping a clear cornea. In vivo, human corneal endothelial cells (HCEC) are held in a non-replicative state within the eye [115].

Although HCEC is not able to actively divide within the eye, ex vivo mechanical wounding studies and treatment of HCEC using EDTA showed that they retain the capacity to proliferate [116]. Therefore, HCEC can proliferate ex vivo and has been successfully used in a first-in-human clinical trial, supplemented with a rho kinase (Rock) inhibitor, by injecting HCEC into the anterior chamber of patients with bullous keratopathy [117].

Regardless, the achieved capacity for establishing a consistent long-term culture of HCEC for ex-vivo corneas or corneal bioengineering is still highly limited due to cellular replicative senescence [116] and endothelial mesenchymal transition [118], so new sources of CECs are, therefore, needed. As CECs have been shown to be derived from the neural crest [119], several lines of investigation have been directed to strategies for differentiation of embryonic and iPSCs into corneal endothelial cells (CEC) [120,121,122,123]. Nevertheless, embryonic cells have several disadvantages, such as an allogeneic origin and the complex regulation involved in the use of such stem cells. In the case of iPSCs, limitations involve the need for retroviral insertion or the use of Sendai virus and oncogenes [27], and the fact that iPSCs are also difficult and time-consuming to establish and culture.

Human ADSCs, on the other hand, can be harvested easily, and are transplantable somatic stem cells. They can be obtained through elective liposuction, and provide a high starting number of cells, with no viral or genetic manipulation necessary and, importantly, a possible autologous use. As previously seen, they are very versatile and can be directed to developing several lineages other than mesenchymal, such as neural crest lineage [124]. In our hands, we tried up to four protocols for inducing the neural crest differentiation from hADSCs and all were successful despite the numerous differences in the culture media’s growth factor composition [27]. In a second phase, to induce CEC differentiation from these previously differentiated neural crest cells, we used the protocols described by Ali et al. [25] and the protocol described by Wagoner et al. [26], for iPSC differentiation into CECs. All the combinations rendered high numbers of Na^+^/K^+^ ATPase-positive cells (at least 80% with each combination or protocol used). In our hands, the Ali protocol was superior, with higher quantitative efficiency (94% Na^+^/K^+^ ATPase-positive cells) [27]. The Ali protocol was the best adapted to CEC differentiation in terms of efficiency, time, and financial cost; however, the Wagoner protocol was superior for CEC marker expression (not only upregulating Na^+^/K^+^ ATPase but also aquaporin and ZO1). Our results broaden the type of cells of autologous extraocular origin that could be employed in the clinical setting for corneal endothelial deficiency, however, the functional performance of extraocular-cell-derived CECs, such as the ADSCs, using in-vivo models remains unexplored. Moreover, transforming MSCs into corneal endothelial cells involves the fact that they need to adopt a quiescent morphology, otherwise, excessive endothelial cell growth could potentially lead to a cellular migration into the angle and iris, and so the risk of subsequent pathology.

## 5. Conclusions

Cellular therapy of the corneal stroma, with either ocular or extraocular stem cells, has been gaining a lot of interest over the last decade. Multiple publications from different research groups are showing their potential benefits in relation to their immunomodulatory properties, capacity to improve or alleviate corneal scars, improve corneal transparency and generate new organized collagen within the host stroma. In contrast to CSSCs or corneal limbal stem cells, autologous extraocular stem cells do not require a healthy contralateral eye and they do not involve any ophthalmic procedure for their isolation. MSCs have been the most widely assayed and they have the potential to differentiate into functional adult corneal cells both in vivo and in vitro, and among them, ADSCs offer several advantages, mainly related to the easy accessibility of their donor tissue and their harvest easiness. While iPSCs are a new exciting source of stem cells, further experience with them is necessary and the costs related to their processing need to be improved. Nevertheless, MSCs and ADSCs secretome (EV’s) is currently being investigated as a potential source for a cell-free based regenerative therapy, in order to obtain equivalent or even better benefits but without providing any cellular component, which may involve the theoretical risk of transferring alive cells with damaged DNA (and so potentially tumorigenic). Despite clinical experience shown in the current review article has not revealed any major safety issues up to date, we shall remember that such studies provide a limited study sample and further trials with larger numbers are still necessary in order to elucidate the precise safety profile and potential risks of such therapies. Thus, since these cellular therapies are very laboratory-dependent, the use of their EVs (potentially enhanced by genetic therapy) could significantly reduce the involved costs, risks and law regulations, allowing for a broader application in real clinical practice.

## Figures and Tables

**Table 1:** MSC differentiation into the different corneal cell lines. Growth factors used in vitro for this purpose, and the markers commonly studied to confirm the correct differentiation into each corneal cell line.

	Growth Factors to Induce Differentiation	Markers Used to Confirm Differentiation
**Corneal Epithelium**	EGF, FGF2, KGF	For epithelium: CK3, CK18, E-cadherin For limbus: ABCG2, p63, CK15
**Corneal Stroma Keratocytes**	FGF2 in keratocyte coculture	Collagens type I, type VI and keratocan
**Corneal Endothelium**	FGF2, Noggin, Heregulin β1, IGF1, PDGFBB, DKK-2	S100, Na^+^/K^+^ ATPase, Cadherin and ZO1

**Table 2:** Clinical trials in which damaged ocular surface epithelium was treated with mesenchymal stem cells (MSC)-based therapy or MSC-derived extracellular vesicle-based therapy. Only clinical trials with published results were included. Abbreviations: ADSC, adipose tissue-derived stem cells; BM, bone marrow; CLET, cultivated limbal epithelial transplantation; DED, dry eye disease; GVHD-DED, dry eye disease associated with graft-versus-host disease; LSCD, limbal stem cell deficiency; MSC, mesenchymal stem cells; NIH, National Institute of Health; OSDI, Ocular Surface Disease Index; UC, umbilical cord.

*ClinicalTrials.gov* Number/Reference	Target Disease	Type of MSC/Route of Administration	Number of Patients	Maximum Follow-Up (Months)	Outcomes after Cell-Based Therapy
Weng et al., 2012 [84]	GVHD-DED	Allogeneic BM-MSC/intravenous injection	22	33	No adverse events Symptoms (OSDI) and signs (NIH consensus criteria, Schirmer’s test) improved in 55% of cases.
NCT01562002 Calonge et al., 2019 [52]	LSCD	Allogeneic BM-MSC/cells cultivated on an amniotic membrane and transplanted onto the ocular surface	27	12	No adverse events The corneal epithelial phenotype improved in patients treated with MSCT (71.4%). Global success was 76.5–85.7% for MSCT at 6–12 months without significant differences with CLET.
NCT03878628 Møller-Hansen et al., 2021 [83]	Aqueous deficient DED	Allogeneic ADSC Transconjunctival injection	7	4	No adverse events Symptoms (OSDI) and signs (osmolarity, TBUT, Schirmer’s test, tendency in corneal staining) improved.
NTC04213248 Zhou et al., 2022 [77]	GVHD- DED	Exosomes isolated from allogeneic UC-MSC/administered as eye drops	14	0.5	No adverse events Symptoms (OSDI) and signs (TBUT, Schirmer’s test, corneal staining) improved.

## Data Availability

Not applicable.
